# Natural Anti-Inflammatory Compounds as Drug Candidates for Inflammatory Bowel Disease

**DOI:** 10.3389/fphar.2021.684486

**Published:** 2021-07-14

**Authors:** Linshan Duan, Shuyu Cheng, Long Li, Yanling Liu, Dan Wang, Guoyan Liu

**Affiliations:** ^1^ School of Pharmaceutical Sciences Xiamen University, Xiamen, China; ^2^ Institute of Gastrointestinal Oncology, Medical College of Xiamen University, Xiamen, China; ^3^ Department of Gastrointestinal Surgery, Zhongshan Hospital of Xiamen University, Xiamen, China

**Keywords:** inflammatory bowel disease, inflammatory, natural anti-inflammatory compounds, drugs, ulcerative colitis (uc), Crohn’s disease (CD)

## Abstract

Inflammatory bowel disease (IBD) represents chronic recurrent intestinal inflammation resulting from various factors. Crohn’s disease (CD) and ulcerative colitis (UC) have been identified as the two major types of IBD. Currently, most of the drugs for IBD used commonly in the clinic have adverse reactions, and only a few drugs present long-lasting treatment effects. Moreover, issues of drug resistance and disease recurrence are frequent and difficult to resolve. Together, these issues cause difficulties in treating patients with IBD. Therefore, the development of novel therapeutic agents for the prevention and treatment of IBD is of significance. In this context, research on natural compounds exhibiting anti-inflammatory activity could be a novel approach to developing effective therapeutic strategies for IBD. Phytochemicals such as astragalus polysaccharide (APS), quercetin, limonin, ginsenoside Rd, luteolin, kaempferol, and icariin are reported to be effective in IBD treatment. In brief, natural compounds with anti-inflammatory activities are considered important candidate drugs for IBD treatment. The present review discusses the potential of certain natural compounds and their synthetic derivatives in the prevention and treatment of IBD.

## Introduction

Inflammatory bowel disease (IBD) is a complex condition with unknown etiology and a tendency of recurrence throughout life. It is a concerning public health issue that is becoming increasingly critical. IBD may be classified into two major pathological subtypes: Crohn’s disease (CD) and ulcerative colitis (UC). UC is a non-specific chronic inflammatory disorder of the rectum and colon, limited mainly to the large intestinal mucosa and submucosa. CD is a chronic inflammatory granuloma, which frequently occurs in the adjacent colon and terminal ileum, although it may affect the whole digestive tract, with a segmental distribution. The etiology of IBD is unclear so far, although the disorder exhibits genetic susceptibility, and its occurrence and development have been associated with immune disorders, environmental factors, and mucosal dysfunction (Park and Jeen, 2019; Larabi et al., 2020; Lee and Chang, 2021). Recent studies have suggested that IBD might be connected with the loss of gut microbial immunological tolerance (Nunes et al., 2018). IBD has become a global disease because of the increasing incidence rate caused by the continuous progress of global industrialization, urbanization, and improving diagnosis (Ng et al., 2017; GBD 2017 Inflammatory Bowel Disease Collaborators, 2020). According to statistics, Europe reports the highest annual incidence rates for CD and UC, 12.7/100,000 and 24.3/100,000 individuals each year, respectively (Molodecky et al., 2012; Mak et al., 2020). Previous studies have shown that the incidence of IBD in Western developed countries tends to be stable (Kotze et al., 2020). Although the incidence of IBD is gradually increasing in Latin America and Asia, the overall prevalence of IBD remains lower compared to the Western world (Kotze et al., 2020).

Currently, the clinical treatment for IBD is mainly based on drugs and surgery, although drug treatment is often accompanied by adverse reactions, which limits the drug usage, and its effect cannot be guaranteed (Rosen et al., 2015; Verstockt et al., 2018). For example, treatment with anti-TNF-α agents increases the risk of opportunistic infection and malignant tumor (Kennedy et al., 2019). Tofacitinib might cause severe infection and increase the risk of malignant tumor (Danese et al., 2018). Surgical treatment also has the risk of complications such as pelvic infection, massive hemorrhage, and intestinal perforation, and it is limited by the age of the patients and physical status (Bhakta et al., 2016). Therefore, it is imperative to develop novel treatment approaches for IBDs. In this context, certain natural compounds and their synthetic derivatives represent a potential research direction and have been adopted in several current research works to unravel their potential in preventing and treating IBD and identify novel candidate drugs for IBD. This paper aims to discuss the present situation of IBD and the natural compounds with the potential to treat IBD.

## Hallmarks of Inflammatory Bowel Disease

### Epidemic Distribution of Inflammatory Bowel Disease in China

According to the existing epidemiological studies conducted in China, Guangdong Province (southern China) has the highest IBD incidence of 1.97–3.44/million individuals each year. In comparison, the Sichuan (Chengdu in southwest China) and Shanxi (Xi’an in northern China) have the lowest IBD incidence of 0.54/million individuals each year (Cui and Yuan, 2018). These data indicate that in China, the incidence of UC and CD is higher in the south compared to the north. Approximately 25% of the IBD patients are aged below 18, and the disease is often more severe in adolescents than in adults. Although China, despite being the most populous developing country in the world, has managed to maintain relatively low prevalence and incidence rates of IBD compared to most of the western nations, an overall upward trend has been observed, with the incidence of IBD rising sharply in recent years (Jiang et al., 2006; Yang et al., 2014a). To date, a national population epidemiological study covering all parts of China is non-existent, and there could be a significantly higher number of IBD patients in China than expected/reported, even higher than the numbers in the western nations (Huang and Aw, 2020). Therefore, vigilance is a must. The current situation of IBD in China is just the tip of the iceberg. Besides China, the prevalence and incidence rates of IBD have demonstrated an upward trend worldwide, indicating that IBD is gradually becoming a global health concern (Kaplan, 2015). According to reports, 80% of the CD patients require intestinal surgery, while over 10% of them require permanent colostomy (Cosnes et al., 2011), which is a great burden on the patients as well as on the whole society (Park et al., 2020).

### Pathogenesis of Inflammatory Bowel Disease

**FIGURE 1:**
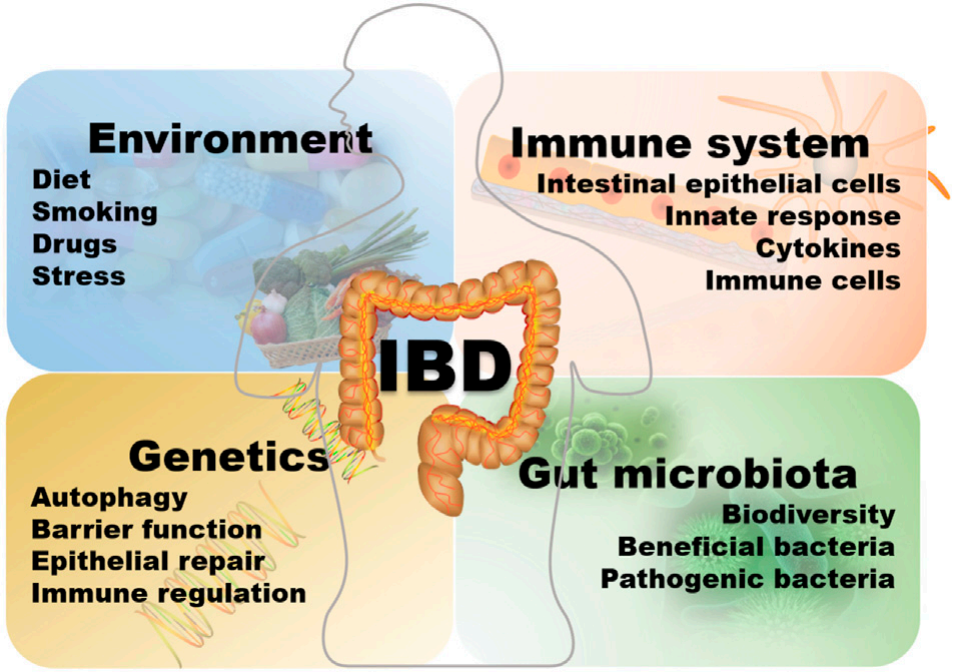
The etiology of IBD.

The etiology of IBD is complex and not completely understood, and could be related to the interaction with the environment, heredity, infection, immunity, and intestinal microorganisms as showed in Figure 1 (Kim et al., 2019). The relevant literature reports multifactorial findings. A detailed understanding of the pathogenesis is necessary for developing an appropriate treatment plan and resolving the limitations and shortcomings of the existing treatment model. The current research on IBD is focused on helper T cells (Th) as previous studies have confirmed that CD is an inflammation condition dominated by Th-1, which produce a large amount of IFN-g and TNF after induction by IL-12, and UC is associated with the Th-2 cells, in which there is a higher production of IL-4, IL-5, and IL-13 while the level of IFN-g is normal (Alfen et al., 2018; Barnig et al., 2019; Bauché et al., 2020; Tindemans et al., 2020). Chronic mucosal inflammation mediated by Th1 or Th2 cells leads to the loss of intestinal wall integrity. It prevents the epithelial barrier regeneration, allowing the intestinal contents, including microorganisms and dietary antigens, to easily infiltrate into the intestine and activate the lamina propria-based immune response, which then leads to IBD. In addition to the classical Th1 and Th2 cell reactions, Th17 cells are reported to have strong correlations with the inflammatory reactions in IBD (Kim et al., 2011; Cătană et al., 2015; Salem et al., 2019). Genetic studies have revealed a certain relationship between the genetic susceptibility of CD and the expression of Th17 cytokines (Patel and Kuchroo, 2015). Th17 cells contribute to the secretion of IL-17 and promote the occurrence of allergic reactions and autoimmunity. Interleukin (IL)-23 is capable of inducing Th17 cells to produce cytokines through the congenital lymphocytes (ILCs) (Cella et al., 2009; Buonocore et al., 2010). IL-17 may induce the production of pro-inflammatory chemokines and cytokines, thereby attracting monocytes and neutrophils at the site of inflammation which promotes the occurrence of inflammatory reactions (Mantovani et al., 2011; Lee et al., 2013; Flannigan et al., 2017; Nadeem et al., 2020). Inhibition of Th17 cells may inhibit the occurrence of inflammation and decelerate the development of colitis. Dendritic cells (DCs) may induce the initial T cells to differentiate into Th1 cells by secreting IL-12, and also produce a large amount of interferon (IFN)-γ to mediate the intestinal mucosal inflammation. The incidence of IBD is closely related to susceptibility genes, such as the IL-23R gene (cWurdinger et al., 2012). Previous studies have revealed that the mechanism underlying IBD pathogenesis involves IL-23R variants. Typically, IBD cases exhibit markedly increased serum IL-23 expression than healthy subjects, which is also positively correlated with UC severity (Neurath, 2019). Since the IL-23 axis is a commonly observed inflammatory pathway in chronic enteritis, blocking the transmission of information through the IL-23/Th17 axis could reduce the development of inflammation (Geremia et al., 2014; Zwicky et al., 2020). In addition, the IL-23R variants are linked with certain extraintestinal diseases associated with IBD, such as psoriasis and ankylosing spondylitis (Brant et al., 2017). Modern treatment of IBD mainly relies on drugs capable of inhibiting these inflammations (Mumolo et al., 2018). Other susceptibility genes related to IBD are NOD2, the autophagy-related genes ATG16L1 and IRGM, IL12B, JAK2, STAT3, CARD15, among others (Lees et al., 2011; Bunte and Beikler, 2019). In addition, Toll-like receptors are strongly associated with the pathogenic mechanism of IBD. Toll-like receptor (TLR) is a pattern recognition receptor that recognizes pathogen-related molecular patterns (PAMPs). Upon activation, TLR is dimerized and then induces a downstream signal cascade reaction to mediate IκB phosphorylation. Consequently, various inflammatory cytokines are produced, leading to the activation of the NF-κB immune signal pathway for excessive immune stimulation. In addition, these cytokines promote Th1/Th2 cell growth and differentiation and modulate dendritic cells (DC) maturation (Chen et al., 2016; Satoh and Akira, 2016; Shi et al., 2019). NOD2, a susceptibility gene, negatively regulates the TLRs and inhibits the overactivation of the NF-κB immune signal pathway. In certain NOD2 variants, this negative regulation might be absent, and the NF-κB immune signal pathway may be abnormally activated, thereby inducing massive Th-1 response and several inflammatory pathways (Schirbel et al., 2019). NOD2 variants may also decrease the ability of the intestinal wall to respond to LPS, which could be related to inflammatory bowel disease (Bonen et al., 2003; Strober et al., 2014). Besides the NF-κB immune signal pathway, the JAK/STAT and the TGF-β1/SMADs signal pathways are involved in the pathogenic mechanism of IBD (Cordes et al., 2020; de Ceuninck van Capelle et al., 2020). In addition, genome-wide association studies have, for the first time, indicated that the pathogenesis of CD might be associated with autophagy (Rioux et al., 2007). Autophagy is a process of cell degradation and recycling, which also contributes to removing intracellular microbes, and is regulated via the lysosomes. Impaired autophagy has been observed in the cells of a series of IBD patients, and several CD-related genetic loci are involved in autophagy homeostasis (Rioux et al., 2007). In 2008, a study evaluated the abnormal miRNA levels in the intestines of UC patients (Wu et al., 2008). Further studies confirmed a possible involvement of miRNAs in IBD via CD68 and NOS2, and also intestinal autophagy through the modulation of the autophagic genes associated with IBD, including ATG16L1, NOD2, and IRGM. It is reported that miRNAs participate in autophagy by regulating the responses of unfolded proteins to the autophagy and IBD-related endoplasmic reticulum (ER) stress, which is achieved via the modulation of the mTOR and NF-κB signal pathways, which allows them to influence pro-/anti-inflammatory effects and inflammatory factors (Sohn et al., 2012; Krissansen et al., 2015; Boros and Nagy, 2019; Arab et al., 2021). The pathways such as NOD2 and autophagy pathways may lead to functional damage in Paneth cells, dendritic cells, macrophages, and absorbent IECs, increasing the risk of developing IBD.

### Therapeutic Targets and Drug-Use Strategy for Inflammatory Bowel Disease

Various therapeutic targets have been approved for IBD. Several attempts have been made to create the next generation of small molecules to be used as specific targets for IBD therapy. For example, in a previous, it was demonstrated, for the first time, that the changes occurring in TGF-β1 in UC and CD are different (Del Zotto et al., 2003). A balanced intestinal immune dynamic relies on the regulation of TGF-β by specific intestinal microorganisms, and abnormal transforming growth factor-β signal has been reported in some instances of IBD, which suggests that TGF-β1 could be used as a therapeutic target in IBD therapy (Ihara et al., 2017). However, several novel methods that could successfully block TNF in RA were ineffective in treating CD (Friedrich et al., 2019), suggesting the possible involvement of additional mechanisms besides simply neutralizing soluble TNF (Levin et al., 2016). In order to develop effective IBD therapies, it is imperative to identify novel potential drug targets and re-examine the existing targets for IBD. The current therapeutic targets for IBD are shown in Table 1.

**TABLE 1:** Therapeutic targets of IBD.

S. No	Target involved	Main origin	Mechanism	Refs.
1.	TNF-α	Macrophages, NK, T, and B cells	Regulate cell differentiation, growth, apoptosis and the production of inflammatory factors and chemokines	Dostert et al. (2019)
2.	IL-12 (IL-12p40)	Monocytes, macrophages, dendritic cells, B lymphocytes and other antigen presenting cells	Regulate the common subunit of IL-12 and IL-23	Feagan et al. (2016), Greving and Towne (2019)
3.	IL-23 (IL-23p19)	Dendritic cells macrophages	Differentiation of immune cells and the production of inflammatory substances	Sarra et al. (2010), Croxford et al. (2014), Feagan et al. (2017)
4.	IL6, IL6R	Macrophages, neutrophils and mast cells	Regulate the inflammatory factors	Allocca et al. (2013), Bertani et al. (2020)
5.	IL-17(IL-17A, IL-17RA)	γδ T cells, ILC3s, invariant NKT cells, and NK cells	Decrease the expression of IL-17 and its receptor	Hueber et al. (2012), Moschen et al. (2019)
6.	IL-10, IL-10R	Virtually all cells of the innate and adaptive immune system, T cells constitute a non-redundant source for IL-10 in many cases	Enhances barrier integrity and regulate the inflammatory response the inflammatory response	Neumann et al. (2019)
7.	IL-13	NK T cells (bearing CD161)	Regulate the inflammatory response	Karmele et al. (2019)
8.	TGF-β1	Immune cells and nonhematopoietic cells	Production of inflammatory factors	Ihara et al. (2017), Kotlarz et al. (2018)
9.	OSM	Produced largely by hematopoietic cells, including T cells, monocytes, macrophages, dendritic cells, neutrophils, eosinophils and mast cells	Promote inflammation	Verstockt et al. (2019)
10.	IFN-γ	NK, T cells	Activation of macrophages and participation in Th1 immune response	Langer et al. (2019)
11.	IL18	Epithelial cells, myeloid cells and lymphocytes	Drive CD4^+^ T cell responses toward a Th1-type and directly mediate inflammation	Williams et al. (2019)
12.	IL1	CD4 T cells	Regulate the inflammatory response	Shouval et al. (2016)
13.	RAR-related orphan receptor	Th17 cells	Differentiation of TH17 cells	Withers et al. (2016)
14.	Caspase-8	Epithelial cells	Apoptosis, necrosis, and inflammation	Becker et al. (2013)
15.	TLR	Immune cells, fibroblasts and epithelial cells	Stimulate downstream signal transduction molecules	Kordjazy et al. (2018)
16.	Defensin(β-defensin)	Intestinal Paneth cells	Trigger of specific immune response	Koeninger et al. (2020)
17.	Smad7	Dendritic cells and CD4T cells	Limit the PDL2/1-PD1 axis	Garo et al. (2019)
18.	JAK (JAK1, JAK3)	T cells, B cells, natural killer cells, macrophages and epithelial cells	Apoptosis, proliferation, migration, development and differentiation of a variety of cell types	Salas et al. (2020)
19.	MAdCAM-1, ICAM-1, VCAM-1	Intestinal high endothelial venules, macrophages, dendritic cells, bone marrow fibroblasts	Recruit inflammatory cells into the peripheral tissues	Allocca et al. (2018), Binder et al. (2018)
20.	Integrin (α4β7/α4β1integrin)	Leukocytes	Mediates leukocyte-endothelial cell adhesion and signal transduction	Sharma et al. (2017a), Danese et al. (2019)
21.	NOD (NOD1, NOD2)	Intestinal epithelial cells and hematopoietic immune cells	Activate inflammatory response	Balasubramanian and Gao (2017)
22.	MDR1	Teff cells	Mitigate oxidative stress and enforced homeostasis in Teff cells exposed to conjugated bile acids (CBAs)	Cao et al. (2017), Ho et al. (2018)
23.	PPAR-γ	Lower intestine, macrophages, and white adipose tissue	Inhibit the transcription of pro-inflammatory mediator genes	Vetuschi et al. (2018)
24.	Ascl2	CD4 T cells	Inhibit the differentiation of Th17 cells and facilitate IL-10 production	Yi et al. (2019)
25.	FcγR (FcγRIIA)	Macrophages	Production of multiple pro-inflammatory cytokines and chemokines	Castro-Dopico et al. (2019)
26.	OCTN1/OCTN2	The 5th chromosome	Maintain physiological cation environments in the organism	Girardin et al. (2012)
27.	HIF-1α-ABC	Th17-cells	Production of pro-inflammatory mediators	Xie et al. (2018)
28.	S1P	Dendritic and endothelial cells	Regulation of the immune system	Peyrin-Biroulet et al. (2017)

In the past decades, extensive research has been conducted to decipher the pathogenesis of IBD, with good results in terms of treatment outcomes. The existing drug treatments for IBD are designed for suppressing massive inflammation and assisting in repairing the digestive tract lining, thereby improving the quality of life of patients with IBD (Wils et al., 2016). The frequently used clinical agents include corticosteroids, aminosalicylates, immunosuppressants, biotherapy agents [different antitumor necrosis factor (TNF) compounds], selective intestinal integrin antagonists, TNF inhibitors, and IL-12/IL-23 inhibitors approved by the Food and Drug Administration (FDA) (Baumgart and Sandborn, 2007). However, these therapeutic agents have certain limitations regarding efficacy, side effects, and administration routes, and their cost is also considerably high. Moreover, long-term usage of drugs or treatments that affect the immune system directly may enhance cancer genesis and progression (Axelrad et al., 2012). If the drug therapy is unsuccessful or the case is complex, surgical intervention is necessary.

European and American guidelines recommend some aminosalicylic acid drugs, such as mesalamine, olsalazine, balsalazide and sulfasalazine, for the induction and maintenance of remission of UC in mild and moderate activity (Harbord et al., 2017). The main adverse reactions are pancreatitis, cardiotoxicity, hepatorenal toxicity and sexual dysfunction (Wang et al., 2016). Corticosteroids (prednisone, hydrocortisone, budesonide, prednisolone, dexamethasone) are mainly used in moderate and severe IBD, especially in acute attacks. Corticosteroids play a decisive role in immunosuppression effects while it also causes severe adverse reactions, such as osteoporosis, mood changes, sleep disorders, which increased the mortality (Berends et al., 2019; Damião et al., 2019). Traditional small molecular immunosuppressive drugs, including azathioprine, 6-mercaptopurine, and methotrexate, inhibit inflammation and inhibit immune response and increase the risk of infection (Broekman et al., 2017; Liu et al., 2017). Currently, the treatment of new biologics for IBD are emerging, including antitumor necrosis factor, anti-adhesion molecule agents, anti-IL-12/IL-23 agents, Janus kinase (JAK) inhibitors. Some novel agents are still under development, becoming the primary option in IBD pharmacological therapies (Chudy-Onwugaje et al., 2019). Tumor necrosis factor inhibitors, among the most commonly used therapeutic drugs, including Remicade (infliximab) and AVX-47091, may improve the clinical scores and mucosal healing in several patients while prolonging the non-recurrence period (Dubé et al., 2015; Andersen et al., 2017). However, patients treated with anti-TNF-α agents will increase the risk of adverse events such as pulmonary tuberculosis and non-melanoma skin cancer (Lichtenstein et al., 2012; Silva et al., 2012; Terdiman et al., 2013). At the same time, tofacitinib and mongersen (GED-0301) are the inhibitors of the Smad7 and JAK signaling pathways (Argollo et al., 2017). The commonly used JAK inhibitors are tofacitinib and filgotinib, in which FDA and EMA have approved tofacitinib to treat moderate and severe UC patients, but there is a black box warning of severe infection and increased risk of malignant tumor in the drug instruction (Olivera et al., 2017). Ustekinumab, aprimod mesylate, risankizumab, brazikumab, mirikizumab are IL-12/IL-23 inhibitors. For instance, aprimod mesylate suppresses the transcription of IL-12/IL-23. In addition, the anti-IL-12ABT-874/J695 monoclonal antibodies may be adopted for targeting the p40 units between IL-12 and IL-23, while SCH-900222 may be used for targeting the p19 subunit specific to IL-23 (Sands et al., 2010). Ustekinumab, an antibody against IL-21 and IL-21, also decreases IL-17 production in the lymphocytes of lamina propria, which inhibits both Th17 and Th1 cells (Fina et al., 2008; Sandborn et al., 2012). In ustekinumab clinical trials, the most common adverse reactions included nasopharyngitis, upper respiratory tract infection, and cough (Davies et al., 2019). Some drugs target the components of the transport pathway, such as MadCAM-1 targeting the adhesion molecules, and anti-α4β7, natalizumab, vedolizumab, and etrolizumab targeting anti-integrin (Moens et al., 2019; Allner et al., 2020). At present, various studies have proved that vedolizumab has good safety in the treatment of IBD, but the high price increases the burden on the patients (Yu et al., 2018a).

It is essential to develop novel therapeutic drugs for IBD. According to recent studies, an increasing number of IBD cases are being reported each day, and in particular, the number of confirmed cases in pediatrics has increased sharply. Moreover, long-term UC and CD are expected to lead to an elevated incidence of intestinal cancer (Bopanna et al., 2017). Parenteral manifestations of IBD are also common, involving the skin, joints, eyes, and kidneys (Bernstein et al., 2001). In addition, there is a higher incidence of other chronic immunological disorders, including primary sclerosing cholangitis, psoriasis, and ankylosing spondylitis (Bernstein et al., 2005). If appropriate measures are not implemented in the next few years, the burden on families and society will increase immensely.

In comparison to western medicine, natural compounds provide the advantages of high efficacy, less adverse reactions, and good tolerance and have, therefore, become the focus of IBD drug research (Khan et al., 2019). In view of the urgent requirement for an effective IBD treatment, the development of novel therapeutics is of significance, and identifying and analyzing the natural compounds exhibiting anti-inflammatory activity may be the appropriate research direction. It would be beneficial to derive lessons from previous research and then focus on developing novel treatments to accelerate the translation of basic research results to clinical application.

## Anti-Inflammatory Natural Compounds as Promising Candidates for Inflammatory Bowel Disease Treatment

Despite the incredible progress of modern medicine, there remain significant obstacles in the path of curing inflammatory bowel disease, which is one of the challenging health concerns for humans worldwide. Novel treatment options for IBD are continuously explored, and novel drugs are being discovered. However, chemically synthesized drugs are always accompanied by adverse reactions. Furthermore, their routes of administration are limited, and their effect cannot be guaranteed. Therefore, in searching for novel pharmacologically active substances that could be used for treating IBD, natural ingredients from various sources, such as plants, animals, and microorganisms, are receiving great attention nowadays. Studies have demonstrated that natural products, including natural medicines, their extracts, and their metabolites, can effectively treat IBD. The section ahead describes these natural products, such as flavonoids, terpenoids, polysaccharides, and alkaloids.

### Flavonoids

**Naringenin** (4',5,7-trihydroxyflavanone flavonoid) is the aglycon of naringin which is abundant in grapefruit (Zaidun et al., 2018; Den Hartogh and Tsiani, 2019). In recent studies, naringenin has demonstrated potential effects in different experimental models of IBD (Pinho-Ribeiro et al., 2016; Zeng et al., 2018). A study reported that naringeninis insoluble in water and soluble in organic solvents as alcohol (Zobeiri et al., 2018). A study demonstrated that naringenin could significantly improve the extent of intestinal edema in a dextran sodium sulfate (DSS)-induced colitis mouse model (Alam et al., 2014). Dou and colleagues reported that naringenin could protect against the DSS-caused colitis in a mouse model. Administration of naringenin before modeling markedly mitigated colitis while promoting monocyte chemotaxis and the colonic secretion of pro-inflammatory factors, such as intercellular adhesion molecule-1 (ICAM-1) and inducible NO synthase (iNOS) (Dou et al., 2013). In addition, the mRNA expressions of IL-6, MCP-1, TNF-α, and cyclooxygenase-2 (Cox2) were decreased. Moreover, the *in vitro* NF-κB reporter assays conducted in naringenin-treated human colonic HT-29 cells revealed that the expression of NF-κB luciferase mediated by TNF-α was significantly suppressed. The above findings are consistent with the results of studies conducted *in vitro*. Naringenin was also demonstrated to protect against colitis in two diverse experimental models. It was reported that naringenin could reduce the expression of PGE2, macrophage inflammatory protein (MIP-2), NO, IL-1b, IL-6, and IL-17A interferon (INF)-c by elevating the colonic mucosal content (Al-Rejaie et al., 2013).

**Catechins** are a kind of polyphenols whose chemical structure have a flavonoid parent nucleus structure, occurring in certain foods and medicinal plants, such as tea, legumes, *Rubiaceae*, *Abarema cochliocarpos*, *Camellia sinensis*, teas, *Mouriri pusa* Garden, buckwheat, grapes, cocoa beans, litchis, and apples (Fathima and Rao, 2016a; Martínez Leal et al., 2018; Cardoso et al., 2020). Catechins mainly include catechin, epicatechin (EC), epicatechin gallate (ECG), (2)-epigallocatechin-3-gallate (EGC) and its stereoisomer gallocatechin (GC), EGCG, and stereoisomer gallocatechin gallate (GCG). Recently, the role of catechins in the prevention and treatment of colitis has been gradually explored. As discovered by Xue et al., EGCG relieved the colitis induced by DSS in the experimental rats. EGCG administered orally at 50 mg/kg BW efficiently reduces colonic mucosal injury and suppresses inflammatory response, consistent with previous findings (Mascia et al., 2010; Sergent et al., 2010; Oz et al., 2013). In addition, EGCG (40 mg/kg BW) also improved the Th1/Th2 balance and diminished the expression levels of TLR4/MyD88/NF-κB pathway-Related proteins (Bing et al., 2017). Increasing studies indicated that the catechins can alter the intestinal flora*.* Bin and colleagues reported that catechin could inhibit Bacteroidetes and Firmicutes’ growth while down-regulated the rate of Bacteroidetes to Firmicutes (Xue et al., 2016). Catechin also has a selective preference for the virulence of bacteria. Fathima A and workers used catechin to treat gram-positive bacteria (*B. subtilis*) and gram-negative bacteria (*E. coli*). They observed that catechin could be more toxic toward gram-positive bacteria (Fathima and Rao, 2016b). Through the continuous deepening of the research on catechins, researchers found that the concentrations of catechins play an essential role when they are applied to intestinal inflammation. Du and colleagues discovered that the low concentrations of EGCG (20 mg/kg/d) tended to have a better effect on DSS-induced colitis compared to high concentrations of EGCG (50 mg/kg/d). EGCG administered orally at 20 mg/kg/d has higher body weight and lower DAI scores (Du et al., 2019). Bitzer et al. also found that when they administered orally at 3.2 mg/ml EGCG containing drinking water, which equates to 480 mg/kg/d (assuming mice drink 15 ml/100 g body weight of water per day), to feed DSS-induced CF-1 mice, the bodyweight loss induced by DSS was significantly accelerated (Bitzer et al., 2016). These findings were consistent with previous findings. To be specific, Mihye Kim and colleagues demonstrated that higher doses of green tea polyphenols could aggravate DSS-induced colitis (Kim et al., 2010). The same results reported by Guan et al. that EGCG (0.1–0.5%), which equates to 150–750 mg/kg/d, exacerbated bleeding and body weight loss (Guan et al., 2012). A clinical trial observed that treat with EGCG is a benefit to UC patients. Twenty UC Patients were treated by 400 mg of EGCG twice daily, after 56 days, the UC disease activity index score of these Patients was reduced. Moreover, EGCG treatment resulted in only minor side effects (Dryden et al., 2013). Therefore, the dose of catechol is the key to its treatment and prevention of colitis, but there are not many clinical reports on the dose suitable for humans, and further studies are needed.

**Hesperetin** (5,7,3ʹ-trihydroxy-4ʹ-methoxyflavanone) is a natural flavanone glycoside, abundant in citrus fruits, including lemons, limes, mandarins, and oranges (Liu et al., 2016; Shirzad et al., 2017; González-Alfonso et al., 2018). Hesperetin represents various pharmacological characters, including anti-inflammatory, anti-oxidative, anti-tumor potentials (Parhiz et al., 2015; Alu'datt et al., 2017; Chen et al., 2019a; Li et al., 2020a). It is reported to alleviate colitis via blocking the intestinal epithelial necroptosis in the DSS-induced experimental mice model (Zhang et al., 2020). Mayada G and workers reported that hesperetin (50 and 100 mg/kg, p.o.) could improve TNBS-induced colitis symptoms in the experimental rat model, including body weight changes and macroscopic colon damage. In addition, hesperetin also significantly decreased the protein expression of p-JAK2 and p-STAT3 while significantly increased the protein expression of SOCS3 (Elhennawy et al., 2021). As discovered by Fatin et al., hesperetin administered at 100 mg/kg, p.o orally, protected against TNBS-induced colitis in the experimental rat model. It could significantly reduce the levels of MPO, MDA, and pro-inflammatory (Polat et al., 2018). The outcomes were consistent with the results of He et al. (2019a). Hesperetin, as the aglycon of hespeprtin, also possesses an anti-inflammatory character (Guazelli et al., 2021). Clinical data regarding the treatment of colitis have not been accurately studied, but researchers used orange juice, which contains abundant hesperetin, to evaluate its effect on the healthy. A report suggested that the plasma concentration of flavonoids is relatively high after taking orange juice. Then, people who regularly consume orange juice maybe have considerable health effects (Erlund et al., 2001).

**Genistein** (4′, 5, 7-trihydroxyisoflavone) is a plant-derived isoflavone, which is well-known. The sources of genistein are soy-based foods, such as soy cheese, soy drinks, soy milk, and soy-based beverages (Ganai and Farooqi, 2015; Sahin et al., 2019). A study observed that mature soybeans constituted 5.6–276 mg/100 g of genistein (Sefrina et al., 2020). Some plants and foods have also been demonstrated to contain genistein, cauliflower, clover sprouts, barley meal, broccoli, sunflower, caraway, and clover seed (Spagnuolo et al., 2015). An increasing number of reports suggested Genistein possesses favorable pharmacological properties in IBD. Zhang and colleagues added 600 mg genistein/kg diet in the daily diet to feed DSS-induced colitis BALB/C mice for 7 days. They found that colonic inflammatory, inflammation, and gut dysfunction could be relieved by feeding with genistein (Zhang et al., 2017a). Abron and workers also demonstrated that genistein could relieve the colitis induced by DSS in the experimental mice. Genistein could promote the conversion of M1 macrophage to M2 and reduced the inflammatory cytokine levels, such as IL-6, TNFα, MCP-1, and IL-1β. Notably, The above study indicated that in a pilot experiment, they found that the 10 mg/kg body weight dose of genistein used in this study was more efficacious in reducing colitis progression than were other tested doses at 5, 20,40, or100 mg/kg (Abron et al., 2018). However, Yu et al. suggest that when genistein is used at doses 5, 15, 45 mg/kg BW to feed DSS-induced colitis BALB/C mice for ten days, the genistein at 45 mg/kg BW should be given priority consideration (Chen et al., 2019b). No clinical studies have been found to have evaluated the appropriate dosage and drug administration time of genistein to benefit colitis patients. However, 16.7 mg genistein given to postmenopausal women with associated vasomotor symptoms was found to be a safe dosage without any adverse effects (Schneider et al., 2019). Thus, the specific dose and drug administration time of genistein to reduce colitis must be further confirmed.

**Anthocyanin** is a kind of water-soluble natural pigments widely found in plants and foods, such as grape seeds, red grapes, saskatoon berries, *Lycium ruthenicum*, Murray, blueberries, blackberries, purple sweet potatoes, and purple cabbage. It is colored aglycones obtained from the hydrolysis of anthocyanins (Jaakola, 2013; Sui et al., 2016; Silva et al., 2017). Some Anthocyanins can be absorbed from the stomach and the intestines; the others can reach the large intestine in significant amounts (Fang, 2014). A growing body of evidence confirms that regular intake of anthocyanins could benefit human health (Chun et al., 2007; Del Rio et al., 2010; Sharma et al., 2017b). Anthocyanins could modulate the gut microbiota in improving various diseases (Thumann et al., 2019; Tian et al., 2019; Khan et al., 2020). A report suggested that anthocyanins from the fruits of *Lycium ruthenicum Murray* to feed mice at 200 mg/kg/d for days 7 days could reverse the decreases in the relative abundances of *Porphyromonadaceae, Rikenellaceae,* and *Prevotellaceae* induced by DSS (Peng et al., 2019). In a report by Wu et al., anthocyanins, extracted from blueberry, were administered orally at 10, 20 and 40 mg/kg BW and showed positive effect on TNBS-induced experimental mice (Wu et al., 2011). Anthocyanin extracted from black rice and bilberries showed almost identical efficacy in relieving colitis (Piberger et al., 2011; Zhao et al., 2018). The difference is that the anthocyanins from different sources are not unique in the dose of administration in the experiment colitis mice. In clinical study, a 50 mg/subject anthocyanin dose was given to healthy volunteers and had no side effects (Nomi et al., 2019).

**Farrerol** is a 2,3-dihydroflavonoid extracted from *rhododendron*. Farrerol is reported to exhibit various biological effects, such as anti-inflammation, antibiosis, and antioxidation (Lai et al., 2016; Wang et al., 2019a). Xin Ran et al. reported that farrerol could protect against 2,4,6-trinitrobenzenesulfonic acid (TNBS)-induced colitis in a mouse model. Administration of farrerol could significantly improve the body weight (BW) changes, damage to the intestinal epithelial barrier, colon length, and clinical scores, while the generation of inflammatory factors was evidently decreased (Azuma et al., 2013). Farrerol is also reported to protect the LPS-treated RAW264.7 cells. Studies have demonstrated that farrerol significantly reduces the production of inflammatory factors, including TNF-α, IL-6, and IL-1β, by suppressing the phosphorylation of NF-κB, p65, AKT, JNK1/2, and ERK1/2 (Ran et al., 2018).

**Lcariin** (also known as icariin) is the main active ingredient of *epimedium*. It is an 8-prenylflavonoid glycoside compound and can be extracted from the dried stems and leaves of *Epimedium sagittatum*, *Epimedium pubescens*, *Epimedium wushanense*, *Epimedium koreanum*, etc. (Ma et al., 2011; Wang et al., 2018a; He et al., 2020). Tao and colleagues observed that icariin, as the main active ingredient of the Epimedium family, protected against DSS-induced enteritis in mice. Moreover, orally administered icariin remarkably mitigates colitis-related pathological alterations and postpones disease progression. In addition, Oral icariin may inhibit p-STAT1, p-STAT3, and p-p65 expressions and pro-inflammatory factor generation in colonic tissues. Furthermore, icariin is also suggested to suppress T lymphocyte activation and growth in a dose-dependent manner, while suppressing the production of pro-inflammatory factors by activated T cells. Icariin is also reported to suppress the phosphorylation of STAT1 (Th1 transcription factor TF) and STAT3 (Th17 TF) in CD4^+^ T cells (Ci et al., 2012).

**Quercetin** is a frequently used natural flavonoid compound, generally existing in glycosylated forms, such as rutoside (3-rhamnosy-glucosyl quercetin) and quercitrin (3-rhamnosylquercetin) (Tao et al., 2013). Quercetin is reported to alter the intestinal host–microbial relationship through the recovery of the pro-inflammatory, anti-inflammatory, and bactericidal activities of intestinal macrophages, which leads to improvement in colitis (Nogata et al., 2006). Comalada M and colleagues observed that quercetin protected against DSS-induced colitis in the experimental rat model. Quercetin is released when gut microbiota cleave glycosides, which mediate its activity (Caddeo et al., 2014). *In vitro* experiments have revealed that quercetin reduces the bone marrow-derived macrophages’ inflammatory response. Moreover, both *in vivo* or *in vitro* studies have revealed that quercetin inhibits cytokine production and induces NOS through the suppression of the NF-κB signaling pathway while does not affect c-Jun N-terminal kinase (Ju et al., 2018). Dodda et al. verified that 50/100 mg/kg quercetin decreased the TNBS-caused morphological, clinical, and biochemical alterations in rats (Comalada et al., 2005). Another study demonstrated that quercetin could reduce colon damage, regulate MPO activity and MDA levels, and significantly increase the glutathione (GSH) content in a TNBS-induced mouse colitis model (Dodda et al., 2014a). Camuesco D reported that quercetin (1 mg/kg/day) could assist in restoring the inflamed mucosa. In addition, quercetin treatment could improve the DSS-induced colon injury in rats, which was associated with the downregulation of the colonic NOS activity and improved intestinal oxidative stress mediated by the reduction in the iNOS protein expression (Dodda et al., 2014b). Ulcerative colitis organoids are a reliable experimental system with supporting evidence for the positive effect on colitis after administrating quercetin (Dicarlo et al., 2019). In 2010, quercetin supplements were added to the U.S. Food and Drug Administration’s generally recognized safe (GRAS) list as a supplemental ingredient that can be added to foods and beverages up to 500 mg per serving (Food and Drug Administration, 2019).

**Myricetin and kaempferol** are natural flavonol compounds that exist mainly in the form of aglycones in plants. Myricetin is a natural polyhydroxy flavonoid compound extracted from the bark, plant seeds and leaves of *Myrica rubra* (Jones et al., 2011; Imran et al., 2019a; Wang et al., 2019b)*.* The pharmacological activities of these two flavonols, such as antitumor, anti-inflammation, antibiosis, and antioxidation, have been studied extensively (Camuesco et al., 2004). Park et al. reported that kaempferol supplementation in diet mitigated the DSS-induced colitis through a decrease in the biochemical and clinical inflammatory factors, such as IL-6, IL-1b, COX-2, TNF-a, and iNOS, and reduction in the MPO, PGE2, and NO levels in colonic mucosa (Semwal et al., 2016). In addition, kaempferol is reported to upregulate the expression of the trefoil factor family 3 (TFF3) gene, indicating that it exerts a protective effect on the function of goblet cells (Park et al., 2012). The use of 80 mg/kg myricetin was reported to remarkably improve acute UC and upregulate the expressions of TGF-β and IL-10. Myricetin could also evidently elevate the levels of regulatory T cells (Qu et al., 2020).

**Apigenin** is a flavonoid present naturally in several vegetables and fruits, particularly citrus fruits, with grapefruit being particularly rich in its content (Nabavi et al., 2018; Yu et al., 2020). Apigenin possesses excellent antioxidation and anti-inflammation activities and has been recently demonstrated to provide relief in inflammatory bowel disease. Hoensch and colleagues reported that flavonoids could induce protective cytokines and enzymes, resulting in enhanced anti-inflammation through the upregulation of the arylates of hydrocarbon (AH) receptors (Hoensch and Weigmann, 2018). Marquez-Flores et al. demonstrated that apigenin protected against colitis induced by DSS in mice. Moreover, apigenin reduces COX-2, MMP-3, iNOS, TNF-a, and IL-1b expressions by suppressing the inflammasome pathway (Márquez-Flores et al., 2016).

**FIGURE 2:**
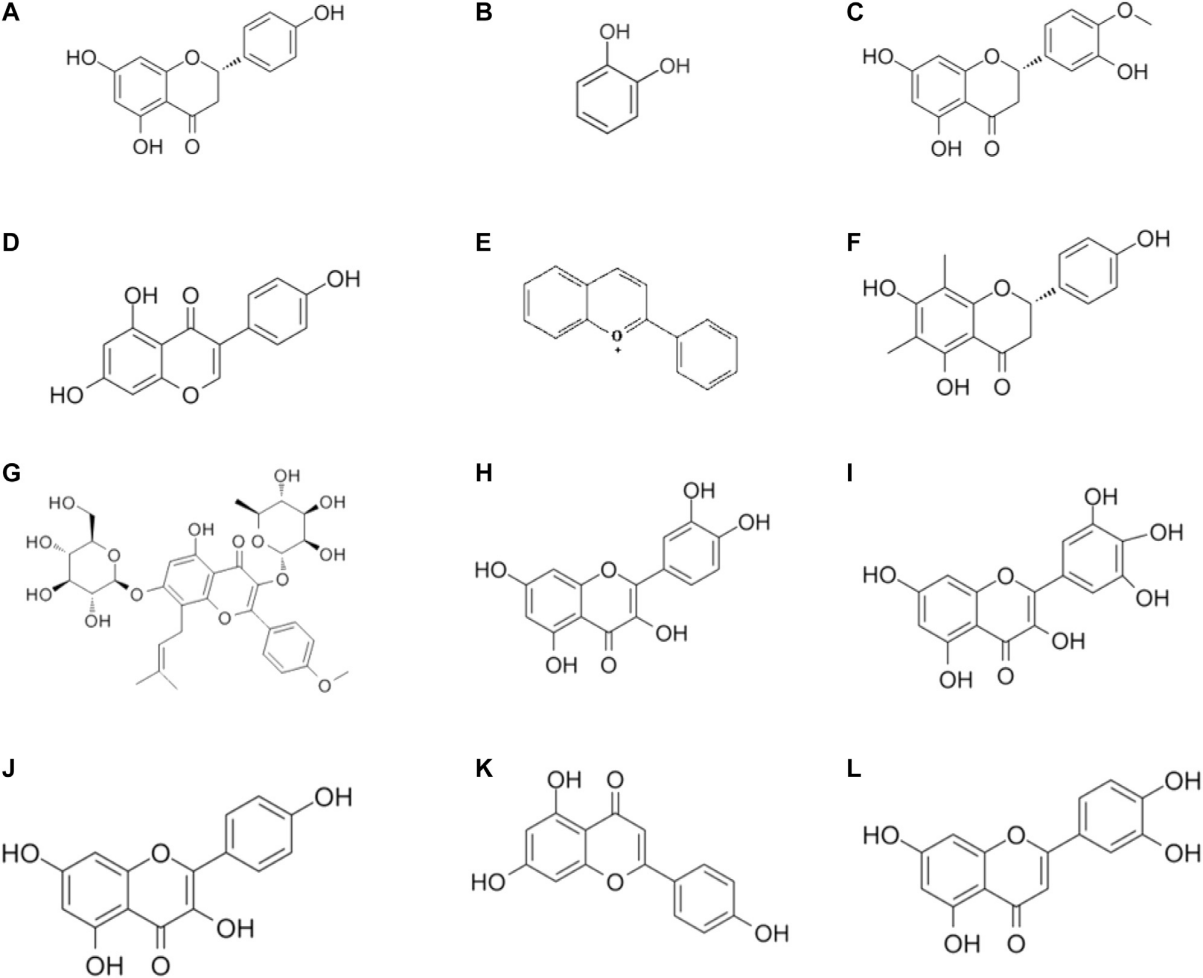
Chemical structures of flavonoid phytochemicals: **(A)** Naringenin **(B)** Catechins **(C)** Hesperetin **(D)** Genistein **(E)** Anthocyanin **(F)** Farrerol **(G)** Lcariin **(H)** Quercetin **(I)** Myricetin **(J)** Kaempferol **(K)** Apigenin **(L)** Luteolin.

**Luteolin** (3',4',5,7-tetrahydroxyflavone) is a common flavonoid that is isolated from celery, honeysuckle, garden bitter melon stems, and other plants across the world (Lin et al., 2008; Aziz et al., 2018; Imran et al., 2019b). Among all flavonoids, luteolin is the one that exerts a significant effect against IBD, as confirmed in various experimental models of IBD. For instance, as reported by Nunes et al., luteolin treatment modulated intracellular inflammatory signaling in HT-29 colonic epithelial cells by suppressing the JAK/STAT pathway (Nunes et al., 2017). Furthermore, this effect was enhanced by administering luteolin in the DDS-mediated colitis mice, probably through the activation of the Nrf2 signaling pathway (Li et al., 2016). According to Nishitani and his colleagues, who conducted both *in vivo* and *in vitro* experiments, luteolin mitigated colitis (Nishitani et al., 2013). An *in vivo* experiment revealed that luteolin protected against colitis in IL-10-deficient (IL-10^−/−^) mice (Karrasch et al., 2007). A recent study suggested that luteolin could affect the intestinal flora, especially the proportion of *Lactobacillus* and *Prevotella-9* (Li et al., 2021). A brief illustration of the different flavonoid phytochemicals is presented in Figure 2.

### Terpenoids

**Ginsenoside** is the major component of *ginseng*. It exhibits immunomodulation and anti-inflammation against IBD and results in an increase in BW and reduction in the colon weight and DAI (Wang et al., 2018b). Notably, the ginseng fruit extract (GB) can suppress the activation of colonic neutrophils, infiltrative T cells, macrophages, and DCs in rats with DSS-mediated colitis. In addition, GB enhances the CD103+CD11c+DCs migration in the colonic tissues of colitis mice while promoting Foxp3+Treg cell differentiation and growth (Zhang et al., 2016a). Fermented red ginseng is reported to inhibit macrophage activity, modulate Th1/Treg cell differentiation, and reduce TNBS-induced colitis (Kim et al., 2018). TGF-β signaling is recognized as a vital anti-inflammatory signal transduction pathway. *In vivo* experimental studies suggest that ginsenosides enhance intestinal mucosal epithelial cell proliferation and modulate the immunocyte differentiation as well as inflammatory factor production, thereby efficiently mitigating the IBD symptoms (Yang et al., 2020). Yang reported that **ginsenoside Rd** could alleviate the TNBS-induced UC symptoms in animals by enhancing the oxidation resistance of injured colons and suppressing neutrophil infiltration (Yang et al., 2012a). On the other hand, ginsenoside Rd is reported to decompose the NLRP3 inflammasome via the AMPK ULK1-p62 axis by means of autophagy and reduce the IL-1β production through the suppression of macrophages, which eventually treats acute colitis in mice (Liu et al., 2018). Ginsenoside Rd is also reported to protect against TNBS-mediated recurrent colitis, possibly through the modulation of p38 and JNK activation, decreasing the expression of the pro-inflammatory factors (IL-1β, IL-6, and TNF-α), and suppressing leukocyte aggregation (Yang et al., 2012b). **Ginsenoside Rg1** (20 (S)-protopanaxatriol) metabolite could inhibit the effect of a combination of LPS and TLR4 on macrophage membrane, recover the balance between Th17 cells and Tregs, and mitigate the inflammatory disorders, including colitis (Lee et al., 2015). It is reported that ginsenoside Rg1 inhibits the production of pro-inflammatory cytokines (TNF-α and IL-1β) by increasing the NLRP2 expression, thereby mitigating the inflammatory reactions in the mice with DSS-mediated colitis. Lee and colleagues used **ginsenoside Re** to treat mice with TNBS-mediated colitis and reported that ginsenoside Re prevented LPS from binding to TLR4 present on the macrophage membrane, thereby efficiently treating inflammation in the blood-brain barrier (BBB) (Lee et al., 2012). **Ginsenoside Rh2** may provide significant relief in the IBD symptoms by activating the activation of the TGF-β signaling pathway and increasing the phosphorylation of the decapentaplegic (Smad) signals from the downstream microblasts. In addition, it may suppress the activation of the pro-inflammatory signal transduction pathways, such as the MAPK and NF-κB signaling pathways (Ye et al., 2014). Tian M and workers observed that **ginsenoside RK3** remarkably relieved the colitis mediated by DSS by suppressing the production of pro-inflammatory factors IL-6, IL-1β and TNF-α (Tian et al., 2020). **Ginsenoside Rb1** administrated by gavaging reduced the expression of pro-inflammatory factors IL-1β, IL-6, and TNF-α, while elevating the expression of the anti-inflammatory factor IL-10 (Joh et al., 2011). According to Li, **Ginsenoside metabolite compound K** mitigated the histopathological outcomes caused due to DSS-mediated colitis in mice, decreased the MPO activity, declined the generation of pro-inflammatory factors IL-6, IL-1β, and TNF-α, and elevated IL-10 expression in the peripheral blood and colonic tissue (Li et al., 2014). Further investigation demonstrated that Ginsenoside metabolite compound K inhibited the production of pro-inflammatory factors within the LPS-activated macrophages through the suppression of the NF-κB signal transduction pathway (Yang et al., 2015).

**Limonin** is a secondary metabolite with high biological activity in plants. It is present mainly in the fruits of Rutaceae plants, such as navel oranges, citruses, oranges, and grapefruits (Zhang et al., 2015; Wang et al., 2018c; Fan et al., 2019). The content in the core (seed) of the fruit is higher, while the content in the peel is low. Studies have demonstrated that limonin significantly reduces the disease activity index (DAI) of IBD, intestinal injury, and pro-inflammatory factor expression. Moreover, limonin remarkably suppresses the production of pro-inflammatory factors in the normal colonic epithelial cells cultured *in vitro*. Furthermore, limonin is suggested to improve UC prognosis by down-regulating p-STAT3/miR-214 (Liu et al., 2019).

**FIGURE 3:**
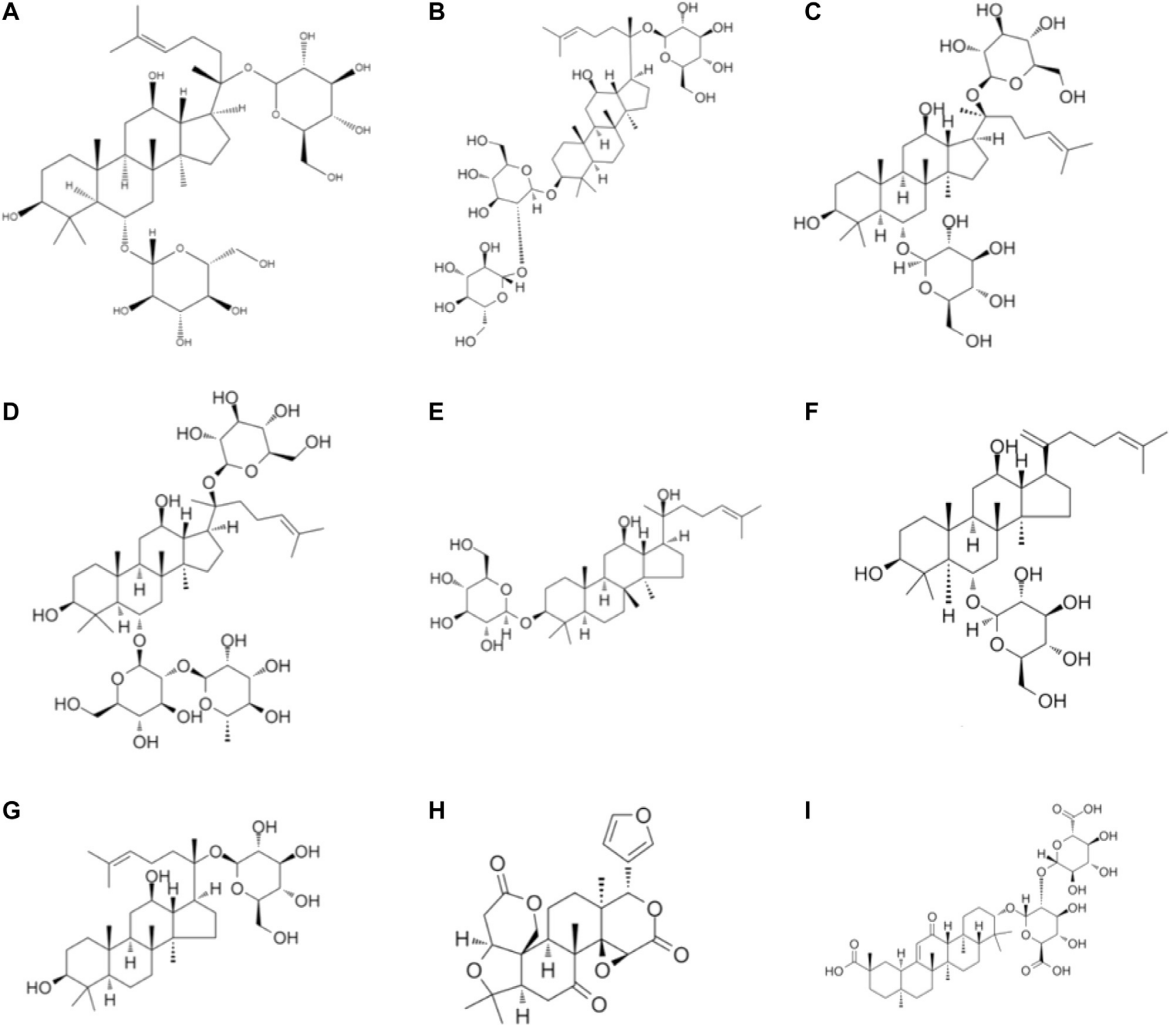
Chemical structures of terpenoid phytochemicals: **(A)** Ginsenoside **(B)** Ginsenoside Rd **(C)** Ginsenoside Rg1 **(D)** Ginsenoside Re **(E)** Ginsenoside Rh2 **(F)** Ginsenoside RK3 **(G)** Ginsenoside metabolite compound K **(H)** Limonin **(I)** Glycyrrhizin acid.

**Glycyrrhizic acid** is one of the active components extracted from roots and rhizomes of *Glycyrrhiza uralensis Fisch., Glycyrrhiza inflata Bat.* and *Glycyrrhiza glabra L*. Glycyrrhizic (He et al., 2019b; Chrzanowski et al., 2021). Liu and workers reported that catechin at 50 mg/kg BW was used to feed TNBS-induced experiment colitis mice and it significantly attenuated pathological changes in the colon. In addition, glycyrrhizic acid (100 mg/ml) reduced the IL-6 production and elevated interleukin-10 production in LPS-challenged macrophages (Liu et al., 2011). The other report suggests that dipotassium glycyrrhizate , a salt of the glycoconjugated triterpene glycyrrhizin, could relieve intestinal inflammation and modify gut mucosal healing. Dipotassium glycyrrhizate could down-regulate the expression levels of gut mucosal healing genes in the acute colitis mice model (Stronati et al., 2019). The solution with glycyrrhizic as the main component has been commercialized. Glycyrrhizin preparation was launched by Minophagen Pharmaceutical (Tokyo, Japan), which has been demonstrated to relieve colitis and decrease the expression levels of pro-inflammatory cytokines and chemokines, including IL-1β IL-6, TNF-α, Cinc-2 (Kudo et al., 2011). Although glycyrrhizic is widely recognized, it still has some limitations. Some researchers observed that glycyrrhizin maybe has side effects, including asthenia and muscle cramps (De Putter and Donck, 2014). A brief illustration of the different terpenoid phytochemicals is presented in Figure 3.

### Polysaccharides

**Astragalus polysaccharide** is extracted from *Astragalus membranaceus* and exhibits various clinical effects (Jin et al., 2014; Auyeung et al., 2016; Zhang et al., 2019). Studies have demonstrated that astragalus polysaccharide (APS) relieves the induction of trinitrobenzene sulfonic acid (TNBS) in colitis rat models. Furthermore, this mitigation effect could be related to the regulation of TNF-α, IL-1β, and NFATc4 expressions (Yang et al., 2014b). Gao and colleagues reported that astragalus polysaccharides promoted the expression of GATA-3 and T-bet, adjusted the GATA-3/T-bet ratio, and exhibited a colonic protective effect in experimental colitis (Gao et al., 2016). Another study demonstrated that the APS treatment could significantly increase BW and improve the DAI and colitis history in a DSS-induced colitis mouse model. Moreover, APS also reduces the NF-ĸВ DNA phosphorylation and downregulates IL-6, IL-17, IL-1β, and TNF-α (Lv et al., 2017). A report demonstrated that APS alone or combined with matrine could alleviate colitis in experimental rats (Yan et al., 2020).

**FIGURE 4:**
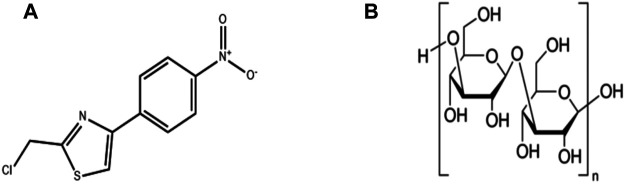
Chemical structures of polysaccharides phytochemicals: **(A)** Astragalus polysaccharide **(B)** Laminarin.

**Laminarin**, a PLS obtained from seaweed, is constituted of the β-(1,3)-linked glucans containing β-(1,6)-linked side chains of lengths and with different distributions (Florez et al., 2017). Rattigan R analyzed the role of chitosan or laminarin supplementation in the diet in the colonic health of DSS-exposed pigs and reported that relative to the baseline DSS group, the kelp DSS group exhibited decreased relative abundances of *Escherichia coli*/Shigella and an increased molar ratio of acetic acid. In addition, kelp was observed to suppress the proliferation of the pathogenic bacteria and increase the content of volatile fatty acids in the colon of the colitis pig model (Rattigan et al., 2020). Rioux L. E., Turgeon S., and Beaulieu M. characterized the polysaccharides extracted from brown seaweeds (Rioux et al., 2010). A brief illustration of the different polysaccharide phytochemicals is presented in Figure 4.

### Alkaloids

**Oxymatrine** is a derivative of matrine n-oxide that is present naturally in *Sophora flavescens* (Xia et al., 2011; Cao and He, 2020)*.* The research on oxymatrine and matrine suggests that oxymatrine at a dose of 63 mg/kg mouse BW remarkably mitigates the colonic damage in the mice with TNBS-mediated colitis, probably through the regulation of the imbalance of Th1/Th2 cytokines. Moreover, the reduction in the colonic IL-2 expression is associated with the increase in the IL-10 levels. Such benefit is associated with the inhibition of p65 NF-kB and b2-adrenergic receptor (b2AR) b-arrestin-2 subunit in the colon and spleen (Wen et al., 2014). Guzman and colleagues studied mice with DSS-mediated colitis and reported that oxymatrine (10 mg/kg BW; i.p.) could block NF-kB's nuclear translocation and activity induced by LPS, thereby improving the overall process of intestinal inflammation, which is unrelated to IkBa degradation/phosphorylation (Guzman et al., 2013). Chen Q and colleagues also observed that oxymatrine (dose: 25, 50, and 100 mg/kg mouse BW; i.p.) could inhibit colonic Th1 and Th17 cell responses via the PI3K/AKT pathway (Chen et al., 2017). Chen F, along with colleagues, constructed a colitis model by administrating a mixture of TNBS and ethanol, followed by oxymatrine [oxymatrine group (20 mg/kg)] seven days after modeling. Consequently, the rats in the oxymatrine group exhibited markedly increased BW, and the length of the colon was also increased significantly. The pathological damage caused to the colonic mucosa had improved markedly. Meanwhile, ICAM-1 and IL-2 expressions had remarkably decreased, in addition to a significant increase in numbers of IL-10, CD11c+, CD103+, and E-cadherin+ cells. Therefore, it was inferred that oxymatrine could increase the number of CD11c+, CD103+, and E-cadherin+ cells by regulating IL-10, IL-2, and ICAM-1, and could also reduce colon inflammation in the experimental rats (Chen et al., 2019c). Certain studies reported that the LD50 value of oxymatrine administered via the intraperitoneal route in male and female mice was 347.44 and 429.15 mg/kg, respectively (Danese, 2012).

**Aloperine** is a tricyclic quinoline alkaloid isolated from *Sophora vulgaris* (Qiu et al., 2020; Wang et al., 2020). As discovered by Fu et al., aloperine protected against the colitis induced by DSS in the experimental mice; to be specific, aloperine administered orally at 40 mg/kg BW efficiently enhanced the DSS-mediated colitis severity, reduced the T cell ratio, and upregulated the Foxp3 levels in mesenteric lymph nodes and spleen. In addition, aloperine (40 mg/kg BW) also inhibits the colonic p-PI3K p85, p-mTOR, and p-Akt expressions in colitis mice while upregulating the protein phosphatase 2A (PP2A) expression. *In vitro* studies have demonstrated that aloperine (0.5 and 1 mM) inhibits the apoptosis of mouse naïve and Jurkat T-cells by suppressing the PI3K/Akt/mTOR signal transduction pathway, although the PP2A inhibitor LB-100 could reverse this beneficial effect. These results suggest that aloperine modulates the colitis-induced inflammatory response by suppressing the PI3K/Akt/mTOR signal transduction pathway via pp2a (Fu et al., 2017; Shi et al., 2018).

**N-methylcytisine** is another tricyclic quinoline alkaloid extracted from *Sophora alopecuroides* and honeysuckle (*Laburnum anagyroides*) seeds (Zhang et al., 2016b; Li et al., 2020b). Its oral administration at the doses of 4 and 16 mg/kg BW markedly mitigated colitis in mice. Moreover, N-methylcytisine could dramatically mitigate the pathological changes and clinical symptoms in DSS-induced colitis mice, which were associated with suppressing colonic pro-inflammatory factors via activating NF-kB by restraining the phosphorylation of IKK and IkB (Jiao et al., 2018).

**Berberine** is a popular alkaloid extracted mostly from Phellodendron and Coptis (James et al., 2011; Zhu et al., 2020). Zhang et al. discovered that oral administration of 100 mg/kg BW of berberine remarkably mitigated the DSS-induced weight loss, shortening of the colon and colon damage. Moreover, berberine promoted intestinal barrier function, anti-inflammatory factor production, and anti-oxidative stress responses, possibly via the STAT3 signaling pathway (Zhang et al., 2017b). In comparison to the model group, berberine could inhibit the expression of IL-1, IL-1β, IL-6, IL-12, TNF-α, TGF-β, and interferon-γ, while upregulating the IL-4 and iL-10 expressions. Moreover, berberine treatment could effectively increase the SIgA level and downregulate MDA and MPO levels. The anti-inflammatory mechanism of berberine hydrochloride is probably associated with suppressing the IL-6/STAT3/NF-κB signal transduction pathway (Zhu et al., 2019). Zhao and colleagues discovered that berberine exerted an effect on the intestinal barrier function of the DSS-mediated colitis mice; besides, berberine at a dose of 100 mg/kg BW markedly suppressed the alterations in the serum expression of zonula occluden-1 (fluorescein isothiocyanate-dextran) and inhibited the expression of epithelial cadherin in colonic tissues (Zhao et al., 2017). Cui, along with colleagues, discovered that berberine at a dose of 40 mg/kg BW (p.o.) decreased gut microbial diversity and interfered with the relative abundances of Bacteroides, Desulfovibrio, and Eubacterium. Berberine treatment in mouse stool transplantation could relieve UC and regulate the balance of Treg/Th17 (Cui et al., 2018). Wang and colleagues reported that berberine markedly enhanced the pathological phenotype of DSS-mediated colitis rats, destruction of the intestinal barrier, and inflammation of the colon. In addition, microbiota analysis based on 16S rDNA revealed that berberine could alleviate intestinal disorders in rats. Berberine is also reported to mitigate DSS-mediated colitis in rats by regulating intestinal microbe-related tryptophan metabolites for activating AhR, thereby greatly improving the impaired intestinal barrier function (Jing et al., 2021). The clinic research observed that berberine administrated at 900 mg/day for 3 months could significantly decrease the Geboes grade of colonic tissue in colitis patients as well as has almost any side effect (Xu et al., 2020).

**Berberrubine** is a natural isoquinoline alkaloid and a major berberine metabolite *in vivo* (Spinozzi et al., 2014). Berberine and berberrubine have been detected in *Rhizoma Coptidis*. The mice receiving berberrubine at the dose of 10/20 mg/kg BW (orders) exhibited markedly alleviated DSS-mediated colitis through a decrease in the DAI and infiltration of the inflammatory cells and the suppression of cytokine generation (IL-4, IL-6, TNF-a, and IFN-g) and the MPO activity. Moreover, it could also upregulate the levels of mRNA adhesion and tight junction (TJ) proteins, including zonula occludens-1/-2, occluding, and claudin-1, while reducing the expression of these proteins reduced the Bax/Bcl-2 ratio. Such benefit is comparable to the use of berberine at a dose of 50 mg/kg mouse BW (p.o.), which suggests the obvious better effect of berberrubine against colitis compared to berberine (Yu et al., 2018b). Yan F, along with colleagues, observed that Berberrubine could suppress the generation of pro-inflammatory factors within the epithelial cells and colonic macrophages in mice while inhibiting apoptosis in colonic macrophages. Berberine also reduces the activation of signaling pathways induced by the pro-inflammatory cytokines MAPK and NF-κB in the macrophages and epithelial cells of the mouse colon treated by the demand side (Yan et al., 2012).

**Caffeine** is a typical naturally occurring xanthine alkaloid. As discovered by Lee et al., exposure to caffeine prevents colitis through a decrease in the expression of chitinase 3-like 1 in the intestinal epithelial cells (IECs). The *in vitro* studies by these authors suggest that caffeine treatment at the doses of 2.5 and 5 mM markedly reduces the mRNA levels of chitinase 3-like 1 in the IEC strain, thereby reducing bacterial invasion in a dose-dependent manner. In addition, compared to the control group mice, the caffeine-treated colitis mice produced fewer pro-inflammatory cytokines, and fewer bacteria entered the other organs (Lee et al., 2014).

**Theophylline** is an alkaloid extracted from tea, which is similar to caffeine in structure and pharmacology (Senchina et al., 2014; Singleton et al., 2019; Zhou et al., 2019). According to a report by Ghassemi and colleagues, theophylline treatment at the doses of 20 and 50 mg/kg mouse BW (i.p.) decreased acetic colitis severity through a decrease in the cytokine levels (IL-6, IL-1b, and TNF-a) and MPO activity in the colon (Ghasemi-Pirbaluti et al., 2017).

**Pentoxifylline** is a xanthine derivative (Annamaraju and Baradhi, 2020). Reyhan and colleagues conducted experiments on rats with ischemic colitis and observed that pentoxifylline administered orally at the dose of 50 mg/kg BW markedly decreased the MDA levels and the ischemic area in the colon (Reyhan et al., 2014). Similarly, Karatay and colleagues reported that pentoxifylline administration at the dose of 100 mg/kg BW affected the TNBS-mediated colitis in rats. According to their results, intrarectal and intraperitoneal administration of pentoxifylline markedly mitigated colonic damage by reducing the MDA levels, as well as the levels of TGF-b1 MPO, MMP-1, and MMP-3 levels, thereby recovering the SOD activity. As indicated by these results, pentoxifylline inhibits colitis partially through the suppression of metalloproteinase activity and oxidative stress. It is noteworthy that caffeine and theophylline also exert toxic effects. The suggested lethal dose of caffeine in the bloodstream is 80–100 mg/ml, which is reached by consuming approximately 10 g or higher of caffeine (Karatay et al., 2017).

**FIGURE 5:**
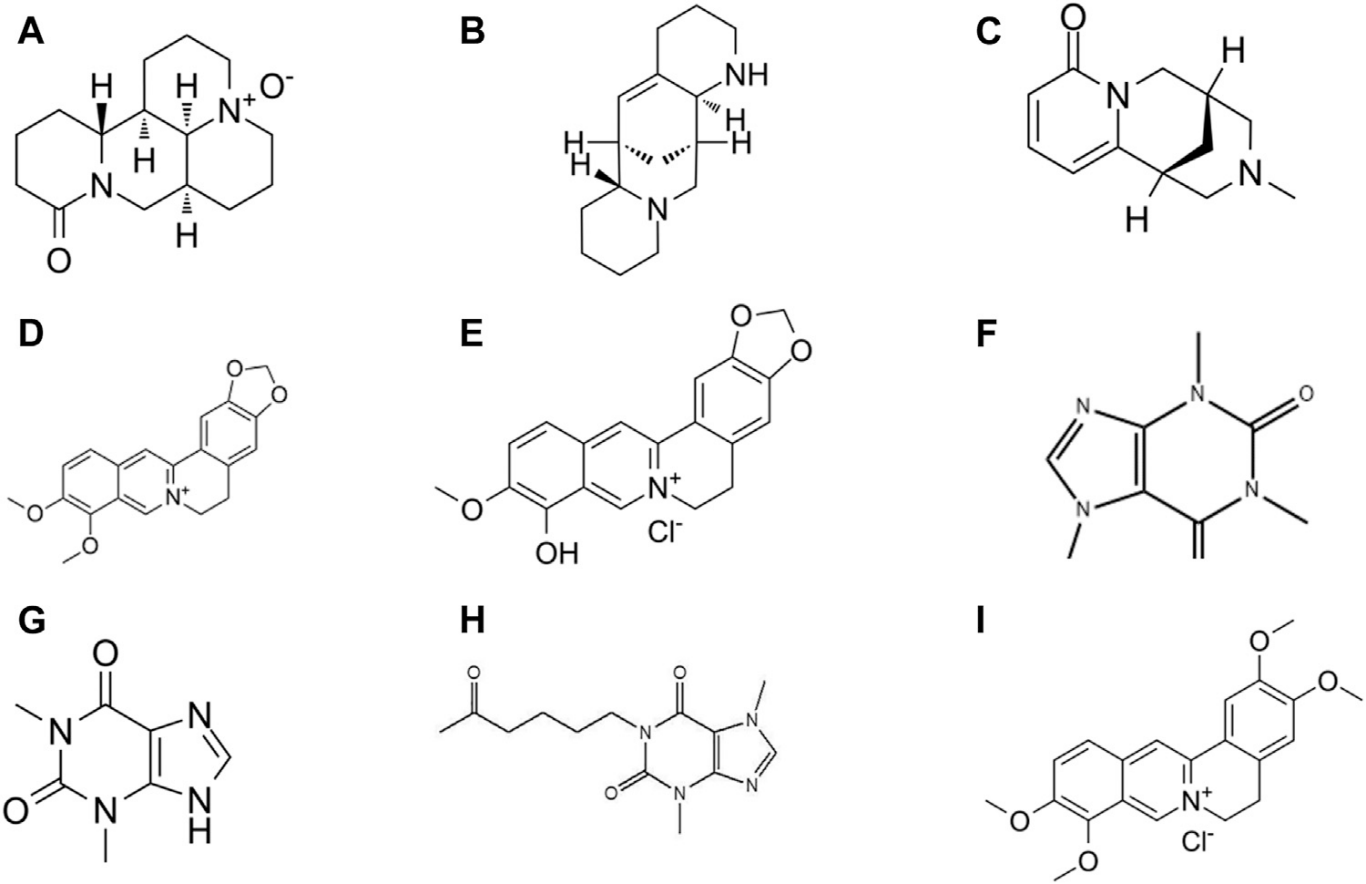
Chemical structures of alkaloid phytochemicals: **(A)** Oxymatrine **(B)** Aloperine **(C)** N-methylcytisine **(D)** Berberine **(E)** Berberrubine **(F)** Caffeine **(G)** Theophylline **(H)** Pentoxifylline **(I)** Palmatine.

**Palmatine** is present in *Phellodendron chinense*, coptis, corydalis, and other plants (Ryuk et al., 2012; Li et al., 2017; Hao et al., 2020). Zhang X. and colleagues reported that the use of palmatine at the doses of 50 and 100 mg/kg mouse BW (p.o.) during DSS-induced mouse experiments improved the integrity of the mucosa and inhibited epithelial cell apoptosis. In addition, palmatine treatment increased the relative abundances of *Firmicutes* and *Bacteroidetes*, which then reduced the bacterial quantity, thereby effectively promoting the recovery of colitis (Zhang et al., 2018). Furthermore, Cyclosporin A is reported to remarkably suppress the efficacy of palmatine in treating DSS mice, which suggested that the anti-colitis effect of palmatine was strongly associated with mitochondrial autophagy (Mai et al., 2019). A brief illustration of the different alkaloid phytochemicals is presented in Figure 5.

## Conclusion

As the pervasiveness of inflammatory bowel disorder across the world is increasing and the available clinical drugs against IBD present side effects and limitations, novel treatments are required. The existing studies explain the pathogenesis of IBD in terms of etiology and pathology. However, there is strong evidence that natural products play vital roles in preventing and treating IBD. In comparison to conventional drugs as alternative treatments, natural products are in high demand as they are safe and effective in treating IBD. A review of the literature revealed that natural components have demonstrated favorable results in various *in vivo* and *in vitro* IBD studies and could be used to treat and avert the progression of IBD. The present report lists and describes the targets reported in the existing research. Moreover, it also reviews the anti-inflammation effects of various natural compounds and their underlying mechanisms in the prevention and treatment of IBD. Natural compounds have positive effects on production of inflammatory cytokines and could protect or relieve colitis. Therefore, natural anti-inflammatory compounds are the first choice when we are developing drugs to treat colitis. In addition, most of the experimental studies have been performed on mice model and the identical compound was used in different dosages to demonstrate its anti-inflammatory activity. However, there is still no clear clinical study to illustrate the optimal dose for colitis patients. Although natural anti-inflammatory compounds have great potential to be developed for treatment of IBD, we need more data to support their safety, dosage and metabolism.

## Glossary

IBD: inflammatory bowel disease
CD: Crohn’s disease
UC: ulcerative colitis
APS: astragalus polysaccharide
Th: helper T cells
TNF-α: Tumor Necrosis Factor alpha
TGF: transforming growth factor
IFN-γ: interferon γ
IL: Interleukin
ILCs: innate lymphoid cells
DCs: Dendritic cells
TLR: toll like receptor
PAMPs: pathogen-related molecular patterns
NF-κB: nuclear factor kappa-B
LPS: lipopolysaccharide
JAK: Janus kinase
STAT: signal transducer and activator of transcription
NOS2: nitric oxide synthase 2
ATG16L1: Autophagy related like 1
IRGM: Immunity-related GTPase family M protein
ER: endoplasmic reticulum
mTOR: mammalian target of rapamycin
IECs: intestinal epithelial cells
FDA: Food and Drug Administration
EMA: European Medicines Agency
MAdCAM-1: mucosal addressing cell adhesion molecule-1
DSS: dextran sodium sulfate
ICAM-1: intercellular cell adhesion molecule-1
iNOS: inducible NO synthase
Cox2: cyclooxygenase-2
MCP-1: Monocyte chemoattractant protein-1
PEG2: prostaglandin E2
MIP-2: macrophage inflammatory protein
OSM: Oncostatin M
VCAM-1: vascular cell adhesion molecule-1
RORs: related orphan receptor
MDR1: multi-drug resistance gene
CBAs: conjugated bile acids
PPAR-γ: peroxisome proliferators-activated receptors-γ
Ascl2: achaete-scute family BHLH transcription factor 2
OCTN: Recombinant Organic Cation/Ergothioneine Transporter
HIF-1: hypoxia inducible factor-1
S1P: sphingosine-1-phosphate
EC: epicatechin
ECG: epicatechin gallate
EGC: (2)-epigallocatechin-3-gallate
DAI: disease activity index
SOCS3: Suppressor of cytokine signaling 3
TNBS: 2,4,6-trinitrobenzene sulfonic acid
MPO: Myeloperoxidase
MDA: malondialdehyde
BW: body weight
